# Biases affecting injected doses of an experimental drug during clinical trials

**DOI:** 10.1186/s13063-016-1463-5

**Published:** 2016-07-16

**Authors:** Nancy Perrottet, Françoise Brunner-Ferber, Eric Grouzmann, François Spertini, Jérôme Biollaz, Thierry Buclin, Nicolas Widmer

**Affiliations:** Service of Pharmacy, Lausanne University Hospital, Lausanne, Switzerland; Brunner Naga, Health Science Consulting, Pfäffikon-ZH, Switzerland; Division of Clinical Pharmacology, Lausanne University Hospital, Lausanne, Switzerland; Division of Immunology and Allergy, Lausanne University Hospital, Lausanne, Switzerland

**Keywords:** Clinical trials, Phase I as topic, Drug evaluation, Pharmacokinetics, Bias, Drug dose

## Abstract

**Background:**

During clinical trials, researchers rarely question nominal doses specified on labels of investigational products, overlooking the potential for inaccuracies that may result when calculating pharmacokinetic and pharmacodynamic parameters. This study evaluated the disparity between nominal doses and the doses actually administered in two Phase I trials of a biosimilar drug.

**Methods:**

In Trial A, 12 healthy volunteers received various doses of an interferon β-1a biosimilar via either subcutaneous or intravenous injection, prepared by partially emptying 0.53 ml syringes supplied by the manufacturer. In Trial B, 12 volunteers received three different formulations of the drug via intravenous injection (biosimilar with and without albumin and a comparator), followed by multiple subcutaneous injections. In both trials, the dose administered was calculated as *D = C* × *V* − losses, where *C* is the drug concentration assessed using ELISA, *V* is the volume administered calculated using syringe weighing and losses are deduced from *in-vitro* experiments. Interferon binding to added albumin and infusion lines was evaluated using a ^125^I-interferon tracer with gel-filtration chromatography.

**Results:**

In Trial A, measured concentrations were close to the nominal strength indicated by the manufacturer (median bias: −6 %), whereas in Trial B they differed significantly for all three formulations (median biases: +67 %, +73 % and +31 % for the biosimilar with albumin, the biosimilar without albumin and the comparator, respectively). In Trial A, the doses actually administered showed large variability and biases, especially at the lowest doses. Indeed, actually injected volumes differed by as much as 74 % from theoretical volumes – a phenomenon mainly attributed to unnoticed fluid re-aspiration through the syringe needle. This was corrected in Trial B. Interferon was not significantly adsorbed on the infusion lines used for intravenous administration. Its binding to albumin was slow, reaching 50 % after a 16 h incubation.

**Conclusions:**

These examples illustrate the importance of assessing the actual doses administered in clinical trials, to ensure accuracy in the determination of clearance, distribution volume, bioavailability and dose–response relationships.

**Trial registration:**

Clinicaltrials.gov NCT02515695 (Trial A) and NCT02517788 (Trial B). Registered on 24 July and 5 August 2015, respectively.

## Background

Over the past few decades, the assessment of a new drug’s pharmacokinetic properties has become a recognized prerequisite for its rational use in therapy. Pharmacokinetic and pharmacodynamic factors are now understood to be among the commonest explanations of undesired variability in drug response. However, a literature review revealed a striking contrast between the great attention devoted to analytical methods and the mathematical modelling of pharmacokinetic data in clinical trials, and the lack of care paid to the pharmaceutical aspects of drug administration [1]. Many clinical researchers simply assume that the dose is the nominal amount declared on the drug’s label, overlooking noticeable biases that inaccuracies may introduce in the assessment of pharmacokinetic parameters [1]. For example, calculation of clearance (*CL*) depends directly on the dose:

$$ CL=\frac{D}{AUC} $$

where *D* is the dose and *AUC* the area under the plasma concentration-time curve.

We have previously proposed six criteria with which to identify clinical trials that might be especially at risk of an error when measuring drug concentrations in body fluids (Table 1) [1]. A retrospective literature analysis of 193 articles showed that about one quarter of clinical trials met at least three of these criteria, indicating a clear need for more accurate assessments of the doses administered to subjects. However, only 5 % (9/193) mentioned any type of verification of the actual dose administered [1]. Indeed, accurate determination of administered doses is rarely, if at all, included in current trial procedures.

**Table 1:** Criteria for identifying clinical trials that may benefit from accurate determination of doses administered

1	Studies to determine a drug’s absolute clearance or volume parameters
2	Studies in which a drug is administered intravenously
3	Studies using a drug not manufactured according to industrial standards
4	Studies on peptides, proteins or other complex biological agents
5	Studies using immunologically based analytical methods
6	Studies using a parallel-group design, with qualitatively different treatments administered to each group

In a medical care context, such issues may have clinical consequences, and unexpected discrepancies between the expected and measured concentrations in drug infusions are not exceptional [2–5]. Until now, to the best of our knowledge, this topic has never been assessed in the context of a clinical trial for biological drugs, whether original or biosimilar.

This investigation thus aimed to evaluate, pragmatically, the biases related to inaccuracies in the dosing of a new biosimilar drug, administered during two Phase I trials.

## Methods

We performed our pharmaceutical evaluations during two Phase I clinical studies (Trials A and B). Both were randomized trials of a crossover design comparing various injection routes, dose levels and formulations of interferon β-1a. The investigations were double-blinded for dose but not for route. The trials’ objectives were to assess the pharmacokinetic and pharmacodynamic profiles of the new biosimilar drug (including linearity and absolute bioavailability), compare the biosimilar drug with the original drug brand and investigate the possible influence of albumin added to the formulation. All participants gave informed consent in accordance with the principles of the Declaration of Helsinki. The study protocols, informed consent forms and related documents were approved by the Human Research Ethics Committee of the Canton Vaud (Lausanne, Switzerland) and by the Swiss Agency for Therapeutic Products (Swissmedic, Bern, Switzerland).

### Trial design

In Trial A, 12 healthy volunteers received single doses, either subcutaneously (1.5, 3.0, 6.0 or 12.0 MIU) or intravenously (0.5, 1.0, 2.0 or 4.0 MIU), of a biosimilar interferon β-1a devoid of human serum albumin (HSA) (Biferonex® HSA-free, manufactured by Rentschler Biotechnologie GmbH, Laupheim, Germany, on behalf of BioPartners, Baar, Switzerland). In Trial B, 12 other volunteers received three different formulations of the drug in crossover by slow intravenous injection (the biosimilar drug devoid of albumin [Biferonex® HSA-free], the biosimilar drug with added albumin [Biferonex® + HSA] and the original comparator [Rebif®, Merck Serono, Geneva, Switzerland]). Subsequently, over 8 days and in parallel, these volunteers received subcutaneous injections every second day, of either Biferonex® HSA-free or Rebif®.

### Study drugs

In both trials, the manufacturer supplied biosimilar Biferonex® as an aqueous isotonic buffered solution of interferon β-1a 6.0 MIU/0.53 ml (overfill of 0.03 ml), stabilized with methionine (pH = 6.8). The pre-filled sterile syringes were equipped with a sealed needle and a protective sheath, ready for subcutaneous injection. This was the planned form for commercialization. In Trial B, the comparator solution (Rebif®) was provided in pre-filled sterile syringes of 6.0 MIU/0.50 ml, ready for subcutaneous administration.

### Dose preparation

In Trial A, a wide range of nominal doses (0.5 to 12.0 MIU) was administered to assess the pharmacokinetic and pharmacodynamic dose proportionalities. As the manufacturer only provided syringes of 6.0 MIU/0.53 ml, the nominal doses were prepared by partially emptying the syringes by drop count. The manufacturer had previously checked that 12 pre-filled syringes delivered a mean (± standard deviation) of 102 ± 4 drops when held vertically (i.e. 1.2 MIU/0.1 ml or 58,824 U/drop). This information allowed us to make an on-the-spot calculation of the number of drops to be removed in order to obtain the target doses.

In Trial B, the dose administered was always 18.0 MIU, corresponding to three 6.0 MIU pre-filled syringes for both Biferonex® HSA-free and Rebif®. For the preparation of Biferonex® + HSA solutions, the contents of three syringes were added to a sterile 5.0 ml glass vial containing 1.6 ml of HSA solution at 30 mg/ml. The preparation was stirred gently to ensure a homogenous mixture without foam formation and left in the vial at room temperature to enable the formation of a stable albumin-interferon complex. Finally, the available volume was aspired from the vial using a 5 ml syringe (subsequently protected by a needle sheath).

### Dose accuracy assessment

**Fig. 1:**
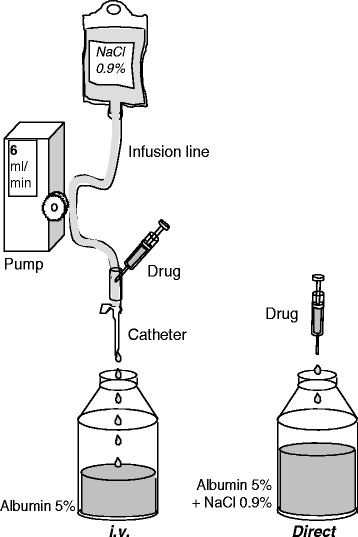
Experiments 1 and 2: in vitro simulation of the intravenous (i.v.) injection process

In both trials, the actual dose administered was calculated as:

$$ D = C\times V-\mathrm{losses} $$

where *C* is the solution’s concentration, *V* the solution’s volume and ‘losses’ represents the amount of drug bound to syringes, infusion lines, containers, etc*.*, as evaluated via in-vitro experiments. Indeed, peptides such as interferon can adsorb onto many types of plastic and glass used for delivery [6, 7], especially in the absence of stabilizing proteins, such as plasma or HSA [8]. We thus decided to validate both the concentration of drug solutions and the procedures for dose preparation and injection. With this intent, two in-vitro investigations (Experiments 1 and 2) were performed prior to clinical trials A and B. A system was designed to compare the amounts of interferon recovered in an albumin/saline solution after direct delivery (i.e. mimicking subcutaneous injection) versus injection through an infusion line (i.e. mimicking intravenous injection; Fig. 1). Human serum albumin was used to imitate the function of blood and to limit drug adsorption onto the flask. Experiment 1 was performed in triplicate at three dose levels (0.5, 1.0 and 4.0 MIU); Experiment 2 was performed in duplicate at a dose level of 18.0 MIU for the three different formulations (Biferonex® HSA-free, Biferonex® + HSA and Rebif®). Any amounts missing after infusion through the catheter and line, compared with direct delivery, would indicate the existence of an adsorption phenomenon along the infusion line and provide a quantitative estimate of the amount of drug absorbed. Any dose-dependency for this adsorption would be shown using ANOVA on log-transformed values to compare relative losses at the different dose levels. During Experiment 1, the solutions were collected in two sorts of tube (type I glass vials and polypropylene tubes, Sarstedt®, Nümbrecht, Germany) to exclude any adsorption on the tube walls and to select the best option for the clinical trials – preliminary tests had shown that polystyrene tubes bound interferon β-1a.

The concentration *C* of interferon β-1a in the solutions used during the trials and in-vitro experiments was assessed using the same immunological assay applied to the clinical pharmacokinetic samples (ELISA kit, BioSource®, Carlsbad, NM, USA). The actual concentrations of Biferonex®, as assessed by the manufacturer using its validated cytopathic assay, was 6.9 MIU/0.53 ml for Trial A and Experiment 1, 6.6 MIU/0.53 ml for intravenous preparations in Trial B and Experiment 2 and 5.9 MIU/0.53 ml for subcutaneous preparations in Trial B.

The administered volume *V* was assessed by systematically weighing all syringes before and after intravenous or subcutaneous administration. To calculate *V*, the weight (*W*) difference:

$$ {W}_{\mathrm{i}} = {W}_{\mathrm{Before}\ \mathrm{i}}-{W}_{\mathrm{After}\ \mathrm{i}} $$

was divided by the solution’s relative density (0.9983 for Biferonex® HSA-free, 0.9871 for Biferonex® + HSA and 0.9950 for Rebif®). Experiments 1 and 2 enabled estimation of the variability of actual doses injected at each dose level. During the clinical trials, the actual weights *W*_i_ injected into volunteers were also compared with the corresponding theoretical weights (*TW*_i_, calculated by multiplying the theoretical volume for injection for a given dose by the relative density) to assess the bias and the variability of the doses administered.

### Profile of drug binding to albumin

Finally, as part of Trial B, a third in-vitro experiment examined whether the binding of HSA to biosimilar interferon β-1a was time-dependent, as a slow binding rate could jeopardize the utility of the resemblance between Biferonex® + HSA and the original comparator, Rebif®. Should biosimilar interferon β-1a remain partly unbound, then it would be more available for binding to target receptors and to subsequent receptor-mediated clearance.

The manufacturer provided an *ad-hoc* solution of the biosimilar drug at a concentration of 0.299 mg/ml (70.3 MIU/ml) in aliquots of 1.67 ml (0.5 mg) containing 4 mM methionine. Interferon β-1a was labelled with ^125^I by ANAWA (Wangen, Switzerland) and prepared in lyophilized aliquots of 2.5 μCi, each containing 25 ng of interferon β-1a. The stock solution of interferon β-1a at 70.3 MIU/ml was diluted to prepare a solution of 12 MIU in a volume of 0.53 ml, which was directly added to the vials containing 2.5 μCi of labelled interferon β-1a.

The experiment involved co-incubating ^125^I-labelled interferon β-1a with HSA at the concentration used for the clinical trial (0.3 %) over different incubation periods (1 h 15 min, 2 h 30 min, 3 h 45 min, 5 h 15 min and 16 h). Rebif® was also used at a concentration of 6.0 MIU in 0.50 ml directly added into a vial containing 2.5 μ Ci of interferon β-1a and incubated for 5 h 15 min and 16 h.

The fraction of interferon β-1a bound to HSA was then separated from the free form using gel-filtration chromatography. With this intent, 25 μl of co-incubation mixture was injected onto a fast protein liquid chromatography column Superose 12 (Pharmacia, Uppsala, Sweden) at a flow rate of 0.6 ml/min using a phosphate buffered saline mobile phase. Fractions were collected every 2 min and radioactivity was measured using a gamma counter (LKB, Mt Waverley, Australia).

Finally, a dose of ^125^I-labelled interferon β-1a was injected through the infusion catheter and line (such as in Fig. 1) to confirm the findings of Experiments 1 and 2.

## Results

### Losses during syringe manipulation and intravenous injection

Experiments 1 and 2 both showed that interferon β-1a was not significantly adsorbed on the infusion line and catheter used for intravenous administration, and was only marginally adsorbed to the syringe (see Tables 2 and 3). In Experiment 1, no losses were induced by collection in either polypropylene tubes (Sarstedt®) or type 1 glass vials (data not shown).

**Table 2:** Experiment 1: losses after intravenous injection of Biferonex®, relative concentrations and actual doses after direct subcutaneous injection at various dose levels

Dose level [MIU] (Biferonex® HSA-free)	Losses after intravenous injection		Relative concentration (direct subcutaneous injection)		Actual dose [MIU] (direct subcutaneous injection)	
	Median	Range	Median	Range	Median	Range
0.5	−4.3 %	22.3 %	93 %	34 %	0.57	0.13
1.0	−10.6 %	31.9 %	115 %	29 %	1.10	0.02
4.0	−7.3 %	15.6 %	94 %	16 %	4.38	0.10

Losses observed after injection through the infusion line (intravenous) versus direct injection (subcutaneous) of Biferonex® HSA-free at different dose levels. A negative value represents an apparent gain. Relative concentrations are expressed with reference to nominal levels

**Table 3:** Experiment 2: losses after intravenous injection, relative concentrations and actual doses after direct subcutaneous injection of different formulations of interferon β-1a

Formulation	Losses after intravenous injection		Relative concentration (direct subcutaneous injection)		Actual dose [MIU] (direct subcutaneous injection)	
	Median	Range	Median	Range	Median	Range
Biferonex® HSA-free	5.6 %	0.6 %	167 %	3 %	18.01	0.02
Biferonex® + HSA	13.6 %	6.5 %	173 %	8 %	16.74	0.05
Rebif®	5.7 %	9.1 %	131 %	19 %	18.33	0.01

Losses observed after injection through the infusion line (intravenous) versus direct injection (subcutaneous) of three different formulations of interferon β-1a at a dose of 18.0 MIU. Relative concentrations are expressed with reference to nominal levels

An injection of ^125^I-labelled interferon β-1a through the same catheter and line confirmed the recovery of 96 % of the radioactivity, whereas 3.6 % and 0.4 % remained bound to the syringe used for administration and to segments of the line, respectively.

### Drug solution concentration

In Experiment 1, the concentration experimentally determined using ELISA was remarkably close to the manufacturer’s declared value (median relative concentration 94 %, with a range of 47 %, see Table 2). In contrast, the concentrations determined in Experiment 2 differed significantly from the manufacturer’s declaration, with a median relative concentration of 168 % (range 12 %) for Biferonex® (with and without HSA), and of 131 % (range 19 %) for Rebif® (Table 3).

### Dose accuracy

In vitro, for a nominal dose of 0.5 MIU, the median actual dose of Biferonex® HSA-free, assessed using syringe weighing, differed by more than 10 % (although not at higher doses, see Tables 2 and 3). In Experiment 2, for a theoretical level of 18.0 MIU, the median actual doses of Biferonex® + HSA and Rebif® were 16.7 MIU and 18.3 MIU, respectively.

In Trial A, the median relative biases in the weight of Biferonex® solution injected into the volunteers ranged from −74 % to +6.1 % (at dose levels of 0.5 and 6.0 MIU, respectively). Median relative biases were typically negative below 4.0 MIU and positive above (Table 4). The median relative biases evaluated during Trial B, using three formulations of interferon β-1a at 18.0 MIU, ranged from −4.2 % to 7.5 % (Table 5).

**Table 4:** Trial A: bias and variability in the amount of injected Biferonex® at different dose levels

Dose level [MIU]	*TW*_i_	*W*_i_	Median relative bias		Route
	[mg]	[mg]	Bias [%]	Range	
0.5	41.6	20.8	−73.6 %	205.1 %	Intravenous
1.0	83.2	50.5	−51.0 %	119.8 %	Intravenous
1.5	124.8	106.5	−15.1 %	30.3 %	Subcutaneous
2.0	166.4	154.3	−8.1 %	23.9 %	Intravenous
3.0	249.6	239.7	−5.4 %	9.7 %	Subcutaneous
4.0	332.8	326.2	−1.3 %	11.8 %	Intravenous
6.0	499.2	529.1	6.1 %	4.0 %	Subcutaneous

Biases evaluated during Trial A with Biferonex® HSA-free at each dose level. *TW*_i_ = weight of solution to inject; *W*_i_ = weight actually injected, bias = median of (*W*_i_ − *TW*_i_)/*TW*_i_, with the range covered by individual measures

**Table 5:** Trial B: biases and variability in the amount of injected formulations of interferon β-1a

Formulation	*TW*_i_	*W*_i_	Median relative bias		Route
	[mg]	[mg]	Bias [%]	Range	
Biferonex® HSA-free	1497.5	1593.9	6.8 %	2.7 %	Intravenous
Biferonex® HSA-free	1497.5	1608.4	7.5 %	4.8 %	Subcutaneous
Biferonex® + HSA	3060.0	2918.5	−4.2 %	7.2 %	Intravenous
Rebif®	1497.5	1538.7	2.9 %	2.4 %	Intravenous
Rebif®	1497.5	1548.8	3.6 %	2.9 %	Subcutaneous

Biases evaluated during Trial B with three different formulations at a dose of 18.0 MIU. *TW*_i_ = weight of solution to inject, *W*_i_ = weight actually injected, bias = median of (*W*_i_ − *TW*_i_)/*TW*_i_, with the range covered by individual measures

### Profile of drug binding to albumin

During Experiment 3, the free form of ^125^I interferon β-1a in NaCl solution eluted between fractions 36 and 42 in gel-filtration chromatography. Increasing amounts of radioactivity, corresponding to high molecular weight HSA complexed with ^125^I interferon β-1a, eluted in fractions 6–12 after 1 h 15 min (15 % binding), 2 h 30 min (18 %), 3 h 45 min (20 %), 5 h 15 min (33 %) and 16 h of incubation (46 %). In the presence of Rebif® increasing amounts eluted too after 5 h 15 min (30 % binding) and 16 h of incubation (44 %). The heavier fraction containing radioactivity was confirmed using ELISA as representing immunoreactive interferon β-1a.

## Discussion

This investigation evaluated the different sources of bias that might affect the doses of an experimental parenteral biological drug administered in two Phase I clinical trials.

### Losses during syringe manipulation and intravenous injection

Experiments 1, 2 and 3 revealed no significant losses of interferon β-1a to syringes and infusion lines in intravenous administration. Nor was interferon β-1a significantly adsorbed onto Sarstedt® polypropylene tubes, which could thus be used throughout the clinical trials and during Experiments 2 and 3.

### Concentration of the drug solution

The concentration of Biferonex® HSA-free assessed using ELISA in Experiment 1 was in the range of 15 % of the nominal dose, whereas Experiment 2 showed a 68 % excess. This significant discrepancy was unexpected. Indeed, cytopathic assays only reported modest differences between Biferonex® batches (<10 % with 6.6 MIU for Experiment 1 and Trial A; 5.9 MIU for Experiment 2 and Trial B). It is, however, not surprising that similarities revealed by a bioassay do not translate directly into similarities in an immunoassay. Although an exact concentration for the reference formulation Rebif® was lacking (no certificate of analysis was available), our assessment revealed a 31 % excess of ELISA-based concentration with regard to nominal strength.

### Drug dose accuracy

Experiment 1 revealed some variability in the actual dose administered after ‘drop count’ preparation and showed this method to be rather inaccurate at lower doses – this was despite the fact that this is the manufacturer’s proposed method for the preparation of a wide range of nominal doses (0.5 to 6.0 MIU). The use of drop count preparation during clinical Trial A would thus have resulted in an erroneous estimation of the amounts of the drug actually administered, especially compared with the precise measurement of an injected weight of drug solution. Conversely, in Experiment 2, where the entire volume contained in a syringe was injected, the actual doses of Biferonex® (with and without HSA) and of Rebif® were accurate.

**Fig. 2:**
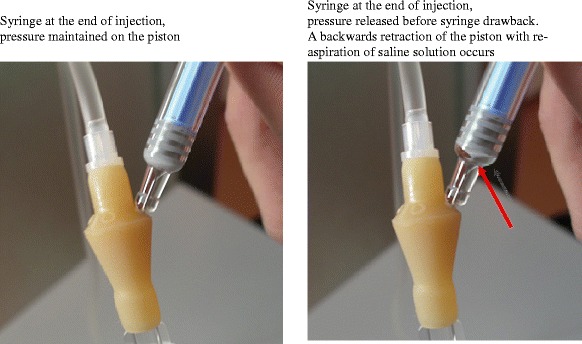
Saline solution re-aspiration after intravenous injection through the infusion line

A further significant issue was identified regarding inconsistencies between the theoretical weights to be administered (*TW*_i_, the product of nominal volume and relative density) and the weights of injected solution actually measured during Trial A (*W*_i_). The median bias was systematically negative for doses ranging from 0.5 to 4.0 MIU, but did not exceed the weight of eight drops (42 mg, i.e*.* 8 % of total syringe volume). At the lowest dose level – 0.5 MIU – administered in this trial, this led to a median relative bias of 74 %, with a huge range of 205 %. Indeed, a thorough examination of the injection procedure revealed that more than 30 mg of aqueous solution could be re-aspired into the syringe from the injection site if no pressure was applied to the piston while removing the needle. This re-aspiration phenomenon is due to the spontaneous re-expansion of the elastic piston in the conic section of the syringe end. During Trial A, our determinations of injected intravenous doses through careful syringe weighing were thus altered by a systematic negative bias resulting from the re-aspiration of a significant volume of saline solution, which mimicked incomplete injections (Fig. 2).

After Trial A, the weight of the re-aspired solution, measured *a posteriori* in all the intravenous syringes (retained in accordance with the rules of Good Clinical Practice), was systematically determined and subtracted from the weight initially determined after injection (*W*_i_). This unexpected finding and its ensuing correction allowed precise doses administered to volunteers to be determined. Conversely, the residual weights quantified in the subcutaneous syringes in Trial A were deemed to correspond mainly to re-aspired drug solution, and were thus considered incomplete injections. Therefore, the weight of subcutaneous injected solution (*W*_i_) was kept unchanged in final dose calculations.

To prevent this bias in the subsequent study (Trial B), the investigators applied a strict procedure to maintain the syringe piston depressed manually under full pressure after injection and until removal of the needle. Consequently, volume-related biases remained limited.

### Profile of drug binding to albumin

Experiment 3 revealed that ^125^I-interferon β-1a binds slowly to albumin, reaching about 30 % binding after 5 h of incubation and almost 50 % after 16 h of incubation. This incubation duration was therefore retained for the preparation of the Biferonex® + HAS solution used in Trial B, as it resulted in a degree of binding close to the comparator, Rebif® (formulated with HSA). The precise molecular interaction between HSA and interferon β-1a is unknown. Taking into account its slow kinetics while adding HSA to the biosimilar probably enabled us to best mimic the reference brand and to avoid potential biases linked to a difference in the free fraction.

## Conclusions

These practical examples, especially regarding biases in actual concentrations (Experiment 2) and volumes injected (Trial A), illustrate that the issue of assessing doses actually administered during clinical trials deserves serious attention, particularly those involving biological drugs. Careful dose determination is essential to ensure accuracy in the assessment of bioavailability, clearance, distribution volumes or pharmacological potency, whether reported in the scientific literature or in drug registration dossiers. Whenever possible, the actual concentrations of the products under investigation, rather than their nominal strength, should be used in calculations, and doses should be corrected for actually injected volumes.

Registration authorities should definitely address the issue of the actual doses administered in their guidance documents on how to carry out Phase I and II trials. Clinical investigators, hospital pharmacists involved in clinical trials and peer reviewers for biomedical journals should all be made aware of this important component of quality management in clinical research.

## References

[1] Buclin T, Perrottet N, Biollaz J (2005). The importance of assessing the dose actually administered in pharmacokinetic trials. Clin Pharmacol Ther.

[2] Allen EM, Van Boerum DH, Olsen AF, Dean JM (1995). Difference between the measured and ordered dose of catecholamine infusions. Ann Pharmacother.

[3] Parshuram CS, Ng GY, Ho TK, Klein J, Moore AM, Bohn D, Koren G. Discrepancies between ordered and delivered concentrations of opiate infusions in critical care. Crit Care Med. 2003;31(10):2483–7. 10.1097/01.CCM.0000089638.83803.B2 14530755

[4] Parshuram CS, Dupuis LL, To T, Weitzman SS, Koren G, Laupacis A (2006). Occurrence and impact of unanticipated variation in intravenous methotrexate dosing. Ann Pharmacother.

[5] Ferner RE, Langford NJ, Anton C, Hutchings A, Bateman DN, Routledge PA (2001). Random and systematic medication errors in routine clinical practice: a multicentre study of infusions, using acetylcysteine as an example. Br J Clin Pharmacol.

[6] Schwarzenbach MS, Reimann P, Thommen V, Hegner M, Mumenthaler M, Schwob J, Guntherodt HJ. Interferon α-2a interactions on glass vial surfaces measured by atomic force microscopy. PDA J Pharm Sci Technol. 2002;56(2):78–89. 11977407

[7] Buchheit KH, Daas A, Jonsson KH (2002). Collaborative study for establishment of an HPLC-method for batch consistency control of recombinant interferon-alfa-2. Pharmeuropa Spec Issue Biol.

[8] Reid RE, Sindelar RD, Lemke TL, Williams DA, Roche VF, Sito SW (2008). Pharmaceutical biotechnology – from nucleic acids to personalized medicine. Foye’s principles of medicinal chemistry.

